# Ketamine Infusion in a Resistant Obsessive-Compulsive Disorder Patient in Bangladesh with Severe Suicidal Ideation: A Case Report

**DOI:** 10.7759/cureus.57877

**Published:** 2024-04-08

**Authors:** Sultana Algin, Debasish Banik, SM Atikur Rahman, Saiful Mahmud Tusher, Fatema Tuj Johora, Asha Akter, Tanbir Ahmed, Md. Abdul Monib Biswas, Susmita Sinha, Mainul Haque

**Affiliations:** 1 Psychiatry, Bangabandhu Sheikh Mujib Medical University, Dhaka, BGD; 2 Anesthesia, Analgesia, and Intensive Care Medicine, Bangabandhu Sheikh Mujib Medical University, Dhaka, BGD; 3 Child and Adolescent Psychiatry, Bangabandhu Sheikh Mujib Medical University, Dhaka, BGD; 4 Physiology, Khulna City Medical College and Hospital, Khulna, BGD; 5 Karnavati Scientific Research Center (KSRC), School of Dentistry, Karnavati University, Gandhinagar, IND; 6 Pharmacology and Therapeutics, National Defence University of Malaysia, Kuala Lumpur, MYS

**Keywords:** y-bocs, bssi, cgi-s, dass-21, compulsions, obsessions, depression, suicidal ideation and attempt, resistant ocd, ketamine

## Abstract

Treatment of resistant obsessive-compulsive disorder (OCD) typically results in insufficient symptom alleviation, and even long-term medication often fails to have the intended effect. Ketamine is a potent non-competitive antagonist of the N-methyl-D-aspartate (NMDA) receptor. Studies have shown that low-dose ketamine infusion results in a considerable reduction in obsessive-compulsive symptoms and a rapid resolution of suicidal ideation. This is a case report on the effect of intravenous ketamine infusion on a patient with resistant OCD and severe suicidal ideation. Intravenous (IV) ketamine was given once a week over consecutive three weeks with necessary precautions. Psychometric tools such as the Yale-Brown Obsessive Compulsive Scale (Y-BOCS), the Clinical Global Impressions Scale (CGI-S), the Beck Scale for Suicidal Ideations (BSSI), and Depression Anxiety and Stress Scale 21 (DASS-21) were applied before and after infusions. Obsessive-compulsive symptoms and suicidal severity started to decrease rapidly after the first infusion. However, after a transient improvement, these symptoms again began to increase after a stressful incident on the second day of the first infusion. All the symptoms measured by validated rating scales showed continued improvement after the following two infusions. The improvement was sustained until discharge (one week after the last infusion) and subsequent follow-up in the sixth and 12^th^ weeks. The role of ketamine in reducing suicidal thoughts and behavior is already established. Very few studies emphasized its effectiveness in improving severe/resistant obsessive-compulsive symptoms. This pioneering work may offer scope for similar research in the relevant field.

## Introduction

Obsessive-compulsive disorder (OCD) is a chronic waxing and waning disorder characterized by recurrent intrusive, upsetting, and unwanted thoughts (called obsessions) and/or ritualized, repetitive behaviors (called compulsions), which are usually performed to reduce the anxiety that the obsessions cause [1-3]. OCD is also considered a neuropsychiatric disease [3]. World Health Organization (WHO) reported that OCD is one of the top 10 most incapacitating illnesses [2]. Around 1.9-3.3% of the global population suffers from OCD [3-6]. One of the considerable complications of OCD is major depressive disorder (MDD) [7-10]. Cai et al. [11] and Lundberg et al. [12] revealed that MDD is significantly associated with suicidal tendencies. Multiple studies have reported that every year, around 700-800 thousand people commit suicide [13-15]. There are 20 attempts of suicide against each case of successful suicide [16].

It has been previously documented that depression can coexist with other psychiatric disorders, including OCD, because of their common psychopathologic characteristics [17-20]. Selective serotonin reuptake inhibitors (SSRIs) are effective in the management of several psychiatric diseases, including OCD and MDD [21-23]. Fernández et al. reported that OCD sufferers have a 10 times higher rate of death by committing suicide than normal individuals [24]. Additionally, among OCD-diagnosed cases, 11.68% attempted to commit suicide and 1.48% resulted in death, respectively [24]. Early diagnosis and intervention of OCD cases have the possibility of good clinical outcomes. Patients often remain symptom-free and enjoy a quality of life [25,26]. It has been reported that from the first development of symptoms to diagnosis and intervention among OCD cases, it often consumes up to 17 years. However, it is a common psychiatric disorder [27-31]. Furthermore, around 50% of OCD cases do not have considerable improvement with psychotherapy [32,33], and 25-50% of cases show poor adherence to pharmacological intervention [33-35]. OCD, when combined with other psychiatric disorders, e.g., MDD or bipolar disorder (BD), aggravates patient situation and it becomes more complex to manage these cases [36-40].

A comprehensive set of randomized clinical trials (RCTs) demonstrated that clomipramine and selective serotonin reuptake inhibitors (SSRIs) (e.g., sertraline, fluvoxamine, fluoxetine, citalopram, escitalopram, paroxetine, sertraline, etc.) were more effective than placebo in the pharmacological intervention treatment of OCD [41-45]. This lays the foundation for clomipramine, and SSRIs are the principal medication for the management of OCD [41-48]. At the same time, many patients with OCD benefit greatly from empirically supported treatments (ESTs) [49,50]. Nevertheless, a significant portion of OCD cases still experience symptoms and impairment even after intensive interventions [26]. Standard and second-line reinforcement therapies remain ineffective for about 40% of OCD patients [51].

Ketamine is a non-competitive, moderately potent, noncompetitive antagonist of the N-methyl-D-aspartate (NMDA) receptor [52], one of the brain's primary glutamate receptors [53]. Glutamate is a vital excitatory neurotransmitter. It is concerned with the cell signaling scheme of the central nervous system [54]. Multiple studies indicate a correlation between OCD and elevated glutamate levels in the prefrontal cortex [55-57]. The "glutamate hypothesis of OCD" was first put forth by Rosenberg et al. [58]. The hypothesis was supported by ﻿Pittenger et al. [59] and Rodriguez et al [60]. Rodriguez et al. demonstrated that a single sub-anesthetic dose of ketamine promptly and vigorously brings down OCD symptoms without giving any SSRIs [60]. Sub-anesthetic dose of intravenous (IV) ketamine has now become a popular treatment option for decreasing OCD symptoms due to its effectual pharmacodynamics in treating suicidal ideation with mood disorders [61,62].

Individuals suffering from OCD are typically aware of their groundless and disproportionate obsessions and compulsive behaviors. Nevertheless, these patients cannot control and resist obsessive deliberations [2]. On many occasions, it is extremely difficult or impossible for OCD individuals to avert their own beliefs and concentrate on work [2]. These patients spend hours contemplating their obsessive ideas. Thus, OCD affects work, study, social relationships, and overall quality of life [3,63,64]. Nagy et al. reported that those OCD cases suffering from high levels of symptoms had a greater tendency to suicidal ideation and attempts [65]. Harris and Barraclough reported in their meta-analysis that suicidal risk among OCD patients is 10-fold higher than among normal individuals [66]. In a systematic review and meta-analysis conducted by Angelakis et al., they observed a statistically significant (Hedges' g=0.66, 95%CI 0.49-0.82) correlation between suicidality and OCD that embraces diverse categories of suicidal thought and endeavor [67]. Similar findings were also reported by multiple recent studies from diverse parts of the globe [68-70]. The purpose of this case report is to assess and validate the effectiveness of IV ketamine infusion in the management of treatment-resistant OCD with severe suicidal ideation. To our knowledge, this is the first case study in Bangladesh that looks at the efficacy of IV ketamine in treating patients with refractory OCD with severe suicidal ideation.

## Case presentation

A 24-year-old, unmarried male from a lower middle socioeconomic background presented to the Department of Psychiatry of Bangabandhu Sheikh Mujib Medical University, Dhaka, Bangladesh, complaining of low mood, disinterest in activities, severe suicidal ideation, and a suicide attempt over the previous two and a half months. He also gave a history of repeated checking and unwanted, intrusive, and distressing thoughts of miscellaneous kinds for the same duration. He was diagnosed as a case of OCD for the last 10 years and has been on treatment for the previous five years. He had been taking fluvoxamine 150 mg with which his symptoms were controlled. After very harsh criticism from his brother, he felt very shameful and reduced the dose of medication. After that, he started having symptoms of depression. At some point, severe suicidal thoughts began, which became so strong that he attempted suicide once by knife two weeks prior to the current presentation and admission. The patient had a history of hospitalization one year earlier when his obsessive-compulsive symptoms became very severe. There was no family history of mental illness and nohistory of substance abuse. He experienced family or domestic conflict during childhood. He had been experiencing stammering since childhood, which became very troublesome in stressful situations. He had taken multiple medications for the last five years, including sertraline, fluoxetine, fluvoxamine, risperidone, aripiprazole, clonazepam, mirtazapine, melatonin, etc. Initially, his compliance and response to treatment were satisfactory. However, his symptoms occasionally got aggravated when he reduced the dose and discontinued drugs due to financial difficulty. Lately, his response to pharmacological intervention (tab fluoxetine 60 mg for 10 weeks) had become suboptimal even with good compliance and adequate dose/duration of medication.

The patient fulfilled the criteria of treatment-resistant OCD (less than 25-35% reduction of obsessive-compulsive symptoms severity measured by Yale-Brown Obsessive-Compulsive Scale (Y-BOCS)) with tab fluoxetine 60 mg for 10 weeks before admission [71]. After admission, a thorough physical examination was conducted, including all vital parameters (pulse, blood pressure, temperature), body weight, and reports of baseline laboratory investigation (complete blood count (CBC), serum glutamic pyruvic transaminase (SGPT), electrocardiography (ECG), serum creatinine) were acquired. A classified anesthesiologist checked and approved physical fitness for anesthetic medication. Then, the patient was shifted to the high-dependency unit (HDU) for ketamine infusion.

Psychometric tools such as Y-BOCS (Table 1), the Clinical Global Impressions Scale (CGI-S) (Table 2), the Beck Scale for Suicidal Ideations (BSSI), and Depression Anxiety and Stress Scale 21 (DASS-21) (Table 3) were applied and recorded before the infusion. The infusion process was methodically monitored through the HDU system. Ketamine infusion started and continued with the presence of a qualified psychiatrist, an anesthesiologist, and two psychiatry residents. The same tools were applied two hours, 24 hours, and 48 hours after the infusion. An identical procedure was followed on three consecutive infusion days.

**Table 1:** Yale-Brown Obsessive-Compulsive Scale (Y-BOCS) Scoring Chart OCD: obsessive-compulsive disorder References: [72,73]

Score	Condition
0-7	Subclinical OCD
8-15	Mild OCD
16-23	Moderate OCD
24-31	Severe OCD
32-40	Extreme OCD

**Table 2:** Clinical Global Impressions Scale (CGI-S) Scoring Chart Notes: In view of the physician's total clinical proficiency with this precise populace, how rationally sick is the patient currently, which is appraised on the succeeding seven-point gauge: 1=normal, not at all ill; 2=borderline mentally ill; 3=mildly ill; 4=moderately ill; 5=markedly ill; 6=severely ill; 7=among the most extremely ill patients [74].

﻿﻿Score	Condition	Symptoms
1	﻿Normal	﻿Not at all ill, symptoms of disorder not present past seven days.
2	Borderline mentally ill	subtle or suspected pathology.
3	Mildly ill	Clearly established symptoms with minimal, if any, distress, or difficulty in social and occupational function.
4	Moderately ill	Overt symptoms causing noticeable, but modest, functional impairment or distress; symptom level may warrant medication
5	Markedly ill	Intrusive symptoms that distinctly impair social/occupational function or cause intrusive levels of distress
6	Severely ill	Symptoms frequently influence disruptive pathology, behavior, and function and may require assistance from others.
7	Among the most extremely ill patients	Pathology drastically interferes with many life functions; they may be hospitalized.

**Table 3:** Depression Anxiety and Stress Scale 21 Scoring Chart Reference: [75]

Subscale	Depression	Anxiety	Stress
Normal	0-4	0-3	0-7
Mild	5-6	4-5	8-9
Moderate	7-10	6-7	10-12
Severe	11-13	8-9	13-16
Extremely Severe	14+	10+	17+

Infusion procedure

The patient received IV infusions of ketamine (0.5 mg/kg) diluted with 100 ml normal saline, by micro burette set over 60 minutes in the HDU [60]. After infusion, the patient was kept in the HDU for eight hours and then was shifted to the psychiatry ward. The patient received a total number of three infusions once a week for three weeks. Each time, the same psychometric tools were applied and recorded as discussed earlier.

Obsessive-compulsive symptoms and severity of suicidal cerebration started to decrease rapidly after the first infusion. After a transient improvement, these symptoms again began to increase after a stressful incident on the second day of the first infusion. Validated rating scales measured all the symptoms and showed continued improvement after the two following infusions. It was sustained till discharge (one week after the last infusion) and over subsequent follow-up at the sixth and 12^th ^weeks.

The patient's obsessive-compulsive symptoms were highly severe (Y-BOCS score: 40) before ketamine infusion. After two hours of the first infusion (immediately after infusion), the score reached 24 (moderate symptoms). It rapidly returned to 36 (extremely severe) after 24 hours and 38 (extremely severe) after 48 hours. This was probably because the patient heard from the college authority that he was not eligible for an upcoming examination. The Y-BOCS score reduced to 28 (severe) immediately after the secondinfusion, 15 (mild) after 24 hours, and 9 (mild) after 48 hours. Score improvement was sustained till the next infusion. It remained subclinical throughout the third infusion and follow-up at the sixth and 12^th^ weeks (Table 4).

**Table 4:** Comparison of symptoms before and after infusions, measured by standard psychometric tools, all of which showed improvement All of those tools showed satisfactory changes in scores from baseline to 48 hours after the third infusion. Changes in obsessive-compulsive symptoms shown by Y-BOCS revealed marked improvements, from 40 (extremely severe symptoms) to 4 (subclinical symptoms). Changes in DASS score revealed a significant reduction of depression, anxiety, and stress (from D-21, A-21, S-21 to D-01, A-0, S-03). BSSI scoring showed a striking reduction in suicidal ideation (from 30 to 3). CGI-S was 7 (extremely ill) at baseline, which came down to 1 (normal), 48 hours after the third infusion. Note: BSSI is a 19-item self-report instrument that measures three magnitudes of suicidal intellection: severity, frequency, and intent. Each item is rated on a scale of 0-2, representing the absence, mild presence, or strong presence of specific suicidal thoughts. The total BSSI score ranges from 0 to 38 [76,77]. O: obsession; C: compulsion; D: depression; A: anxiety; S: stress; Y-BOCS: Yale-Brown obsessive-compulsive scale; DASS: Depression, Anxiety, and Stress Scale; BSSI: Beck Scale for Suicidal Ideation; CGI-S: Clinical Global Impression Severity Scale

Infusion Number	Intervention Time	Y-BOCS	DASS	BSSI	CGI-S
Infusion 1	Before Infusion	40 (O-20, C-20)	63 (D-21, A-21, S-21)	30	07 (Extremely ill)
	2 hours After Infusion	24 (O-16, C-8)	22 (D-15, A-03, S-04)	04	04 (Moderately ill)
	After 24 hours	36 (O-18, C-18)	47 (D-20, A-08, S-19)	24	06 (Severely ill)
	After 48 hours	38 (O-19, C-19)	50 (D-21, A-11, S-18	24	06 (Severely ill)
Infusion 2	Before Infusion	30 (O-14, C-16)	45 (D-17, A-16, S-12)	24	06 (Severely ill)
	2 hours After Infusion	28 (O-12, C-16)	39 (D-17, A-16, S-06)	19	05 (Markedly ill)
	After 24 hours	15 (O-11, C-04)	15 (D-07, A-01, S-07)	07	04 (Moderately ill)
	After 48 hours	09 (O-08, C-01)	03 (D-02, A-0, S-01)	0	02 (Borderline mentally ill)
Infusion 3	Before Infusion	10 (O-06, C-04)	04 (D- 01, A- 01, S-02)	07	02 (Borderline mentally ill)
	2 hours After Infusion	03 (O-0, C-03)	04 (D-01, A-0, S-0)	03	01 (Normal)
	After 24 hours	02 (O-01, C-01)	05 (D-02, A-0, S-03)	03	01 (Normal)
	After 48 hours	04 (O-0, C-04)	04 (D-01, A-0, S-03)	03	01 (Normal)

Regarding suicidality, the BSSI score was 30 (severe) during admission. Immediately after infusion, it came down to 4 (no or minimal ideation). As stated earlier, his stress might have increased the BSSI score after initial improvement. The score (24) remained stationary, considered as severe illness, till the second infusion. Then, it came down to 19 (moderate) two hours after secondketamine infusion, 7 (no or minimal ideation) after 24 hours, and 2 (no ideation) after 48 hours. The score remained minimal throughout the third infusion, discharge, and follow-up period (Table 4).

The DASS-21 score at the time of admission was 63. It started improving following the first infusion: depression: 15 (moderate), anxiety: 3 (normal), and stress: 4 (normal) two hours after ketamine administration. After a transient deterioration (the stressful academic news regarding academic examination), further improvement was observed with a subsequent second and third infusion to a minimum level (3-5). This minimum DASS-21 score was sustained during discharge and follow-up at the sixth and 12^th ^weeks (Table 4).

CGI score was 7 (most extremely ill) before the first dose of ketamine infusion. After two hours, the score was 4, and the patient was moderately sick. In the next 24 and 48 hours, again back score 6 (severely ill). The score level was 6 till the second infusion started. At 48 hours of the second infusion, the score came down to 2. The patient achieved normal level by two hours of the third infusion. The normal level was sustained throughout the third infusion till discharge and the sixthand 12^th^ follow-up periods.

Ensuring compliance

The patient was kept admitted to the Department of Psychiatry for 25 days to ensure compliance. A multidisciplinary team of psychiatrists, psychiatry trainees, anesthetists, and trained nursing staff of HDU supervised the whole procedure. Throughout the procedure, we monitored vital signs, including blood pressure, pulse, respiratory rate, and oxygen saturation. The entire team remained vigilant in every part of the ketamine infusion process for the emergence of any new symptoms, e.g., hypo/hypertension, dizziness, nausea, headache, hallucination, features of dissociation and disorientation, etc., which might happen with ketamine infusion.

Adverse drug reactions

Most of the adverse effects related to ketamine infusion are associated with the anesthetic dose (>2 mg/kg IV) of ketamine, which is much higher than the dose we used (sub-anesthetic dose: 0.5 mg/kg IV). There were no notable adverse effects during or after the infusion except transient drowsiness a few minutes after starting the injection, which lasted for 50-60 minutes and remitted spontaneously without any pharmacological intervention. The most common adverse effects that might happen are disorientation and features of dissociation. We kept the patient admitted for an extended period at HDU to minimize these issues.

Discharge notes

On discharge, the patient was given fluvoxamine 150 mg, duloxetine 20 mg, and propranolol 20 mg to be continued. The patient was advised to follow up every three months or in case of an emergency. Also, the patient was educated and counseled on sleep hygiene, food habits, and lifestyle.

## Discussion

The patient's obsessive-compulsive symptoms were highly severe (Y-BOCS score: 40) before ketamine infusion. The score started reducing after two hours. Nevertheless, the score shot up by 24 hours and became extremely severe by 48 hours. The patient heard about his ineligibility for an upcoming academic examination, which possibly caused despair and stress and contributed to the worsening of his symptoms after initial improvement. The Y-BOCS score again reduced to 9 (mild) after 48 hours of the second ketamine infusion. Score change for the better was uninterrupted till the next infusion. It remained at the subclinical level all through the third infusion and follow-up at the sixth and 12^th^ weeks. The results were in the same line with previous research suggesting that repeated ketamine pharmacodynamics leads to quick anti-obsessional with anti-depressive effects that persist for a considerable amount of time after the medication has cleared [78,79]. Rodriguez et al. reported that ketamine has rapid anti-obsessional effects that carry on for one to seven days post infusion [78]. The ketamine-induced anti-obsessional effect persists even after all medication is cleared from the patient's system. As ketamine is an NMDA receptor antagonist, it modulates the glutamate system among OCD cases [80]. Johnston et al. recently reported that ketamine relieves depression within an hour, not responding to conventional antidepressant medications [81]. This finding has led ketamine to be utilized in neuropsychiatric therapeutics, e.g., "depression, bipolar disorder, anxiety spectrum disorders, substance use disorders, eating disorders, chronic pain, etc.". Ketamine is also useful in anxiety, anhedonia, and suicidal ideation [81]. Bottemanne and Arnould reported that ketamine changes the tone of NMDA receptors and gamma-aminobutyric acid (GABA) receptors, which are principal trails for contingency learning, belief updating, and extinction learning [82]. Another study revealed that ketamine provokes antidepressant pharmacodynamics via augmented neuroplasticity precipitated by an outpouring of glutamate and inflection of GABA. It causes decreased hippocampal GABA+/ total creatine (tCr) ratio two hours following ketamine infusion [83]. 

Regarding suicidality, the BSSI score of the current patient was 30 (severe) during admission. Immediately after infusion, it came down to no or minimal ideation. The patient developed stress because of his academic issues that perhaps increased and deteriorated his BSSI score after the preliminary improvement. The score (24) remained stationary, considered as the severity of illness till the second infusion. Then, it started declining from moderate to no or minimal ideation after 24 hours, and 2 (no ideation) after 48 hours after the secondketamine dosage. Improvement was contained through the full length of the third infusion and follow-up after the sixthand 12^th^ weeks. Many similar earlier studies, including a meta-analysis, corroborate the result that continual sub-anesthetic ketamine infusions significantly and consistently reduce suicide ideation [84,85]. Shivanekar et al. reported that ketamine quickly decreases suicidal thinking by 2-24 hours after a single infusion among patients with intense suicidal thought processes [86]. The antisuicidal effect of ketamine possibly has dissimilar pharmacodynamics to the antidepressant mode of action [87]. Nevertheless, Price and Mathew revealed that ketamine's antisuicidal pharmacological effects are possibly an integral part of its comprehensive antidepressant action [87].

CGI Score was 7 (extremely ill) before the first dose of ketamine infusion. After two hours of the firstinfusion, the score declined, and the patient was improving. However, in the next 24 and 48 hours, the score was again back to 6, which signified the patient was severely ill. The patient's stress might have been triggered because of his academic issues. The score remained at 6 till the second infusion started. At 48 hours of the second infusion, the score came down to 2 (moderately mentally ill). The patient was able to get down to normal level (score 1) by two hours of the thirdinfusion. The normal level remains constant for the whole duration of the third infusion till discharge and the sixth- and 12th-week follow-ups. Similar observations were reported by Singh et al. [88]. Iglewicz et al. reported similarly that repeated administration of ketamine minimizes depression and normalized CGI score [89]. Mandal et al. reported that ketamine has a strong, speedy, and continuous pharmacological effect in the minimization of depression [90]. 

**Figure 1:**
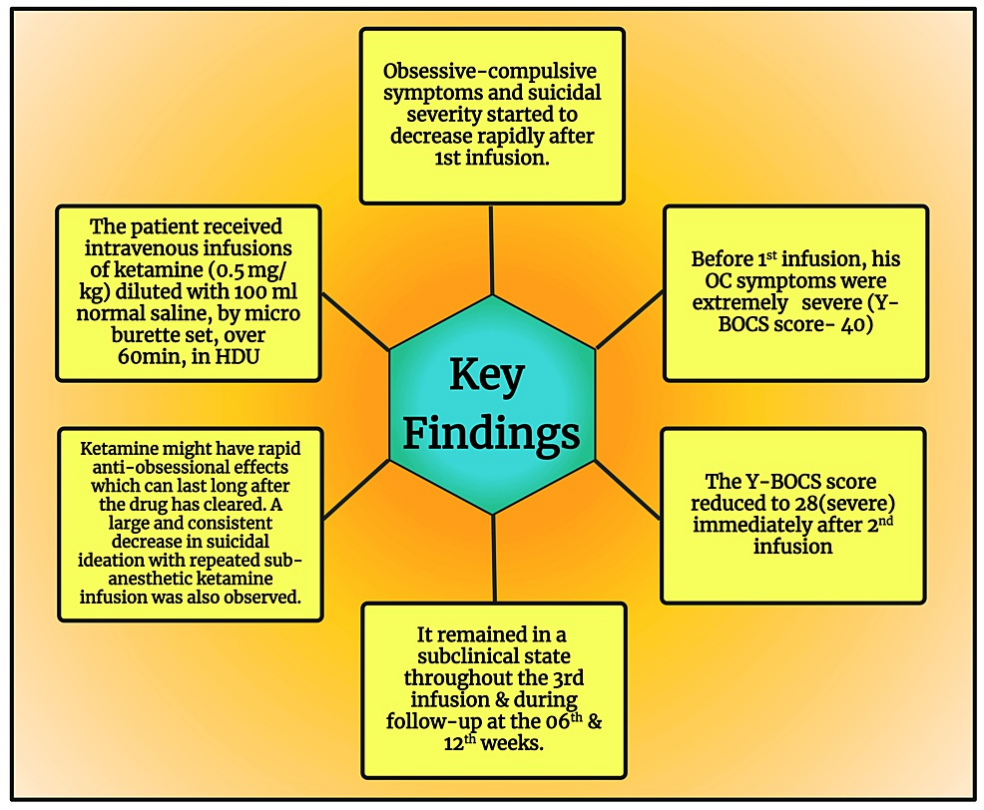
Key Findings of This Case Report Y-BOCS: Yale-Brown Obsessive-Compulsive Scale; OC: obsessive compulsive; HDU: high dependency unit Image Credit: Susmita Sinha; drawn with the premium version of BioRender [96] with the license number YP26LGYQS7

Regarding the minimization of depressive symptoms in the current case, it is in line with the findings of multiple recent research that repeated infusions of ketamine produced a more potent and sustained reduction in depressive symptoms [91-95]. The key findings of this case report are illustrated in Figure 1.

## Conclusions

According to this case report, ketamine has beneficial effects on resistant OCD management. Ketamine minimizes depression, suicidal ideation, and attempts and sustained anti-depressive effects 12 weeks later after the last dose. This report might be used as support in the future for other research to deepen our understanding. We recommend more research among Bangladeshi patients to confirm our data. A multicenter study with reasonable data size is advocated.
